# Reply letter to: If SARS-CoV-2 vaccination is blamed for Parsonage-Turner syndrome, neurosurgical neurolysis is not indicated

**DOI:** 10.51866/lte.46lr

**Published:** 2024-05-23

**Authors:** Zi-Yi Yeoh, Siti Nurkamilla Ramdzan

**Affiliations:** 1 MB ChB, Department of Primary Care Medicine, Universiti Malaya Medical Centre, Kuala Lumpur, Malaysia. Email: yeohziyi@ummc.edu.my; 2 MBBS, MFamMed, PhD, Department of Primary Care Medicine, Faculty of Medicine, Universiti Malaya, Kuala Lumpur, Malaysia.

**Keywords:** COVID-19 vaccines, Brachial plexus neuritis, Primary healthcare

**Dear editor,**

We would like to respond to the letter to the editor titled ‘If SARS-CoV-2 vaccination is blamed for Parsonage-Turner syndrome, neurosurgical neurolysis is not indicated’ addressed to our article titled ‘Parsonage-Turner syndrome: A case report of a rare side effect of COVID-19 booster vaccination’.^1,2^ We thank the author for raising several points worthy of discussion.

In response, we would like to primarily quote the recent review titled ‘Neuralgic amyotrophy: An update on diagnosis, pathophysiology and treatment’ by Van Eijk and colleagues.^3^

First, the pathophysiological mechanism of Parsonage-Turner syndrome (PTS) remains unknown.^3^ The study cited by the author in the letter to the editor is a systematic review of case reports linking PTS with antecedent SARS-CoV-2 infection.^4^ However, it does not establish a definitive association between the two. Similarly, there is also a review of case reports linking PTS with antecedent COVID-19 vaccination, but it does not prove any association.^5^ PTS has been reported after various other infections, such as Coxsackie or hepatitis E viral infection.^3^ Since our patient did not exhibit any viral infection symptoms, the diagnostic value of a virus panel is questionable.

Second, regarding further investigations, patients with PTS may occasionally exhibit elevated levels of cerebrospinal fluid protein and lymphocytes, but their diagnostic value is uncertain.^3^ Researchers have not discovered any potentially causative genetic mutations for PTS, although the role of the *SEPT9* gene mutation is still being researched in hereditary cases.^3^ Additionally, a comprehensive assessment including magnetic resonance imaging (MRI) of the spine and brachial plexus, measurement of fasting blood sugar levels and examination of patient history ruled out neoplastic, diabetic, traumatic and hereditary-related brachial plexopathies. Our original article mentioned the absence of other symptoms, trauma, weight loss, back pain or insect bites as well as the patient’s overall good health without any medical conditions.^2^ The patient’s spinal and brachial plexus MRI revealed preserved right C5-7 nerve roots with no abnormal enhancement or thickening of the brachial plexus. The patient did not consume alcohol, but this detail was not explicitly described due to limitation in the word count.

Third, the treatment of PTS is currently guided by anecdotal evidence, as highlighted in a 2009 Cochrane review cited in our original article.^6^ Reports of successful neurolysis in PTS cases exist.^3,7,8^ A recent review suggests that peripheral nerve surgery, such as radial-to-axillary nerve transfer, may be considered for selected severe cases.^3^ However, further research is needed to establish the routine use of peripheral nerve surgery as a treatment option.^3^

Fourth, visible male breast enlargement can be attributed to true gynaecomastia or pseudo-gynaecomastia, with the latter being more common and caused by excess adipose tissue behind the nipples. Examination revealed that the breast enlargement of the patient was due to adipose tissue.

Lastly, multifocal motor neuropathy was not considered in our patient, as the nerve conduction study did not indicate focal demyelination and conduction block.^9^ As detailed in our case report, the nerve conduction study revealed a preserved conduction velocity with reduced compound muscle unit action potential amplitudes, suggesting the absence of demyelination and indicating possible axonal loss.^2^

We hope that this explanation provides clarification, bearing in mind that the authors are primary care physicians without specialised training in neuromedical sciences.
